# Detection of Anti-Platelet Factor 4 Antibodies in Acute and Chronic Thromboembolic Disease—A Pilot Study

**DOI:** 10.3390/ijms27219899

**Published:** 2026-11-05

**Authors:** Annabell Trunk, Jan Wesche, Stella Jacobi, Marina Bruch-Oms, Donata Metzler, Eva Herrmann, Thomas Thiele, Edelgard Lindhoff-Last

**Affiliations:** 1 Coagulation Centre, Cardiology Angiology Centre Bethanien Hospital (CCB), 60389 Frankfurt, Germany; 2 Coagulation Research Centre Bethanien Hospital, 60389 Frankfurt, Germany; 3 Institute for Transfusion Medicine, University Hospital Greifswald, 17475 Greifswald, Germany; 4 Werfen, 08186 Lliçà d’Amunt, Barcelona, Spain; 5 Goethe University Frankfurt, Institute of Biostatistics and Mathemical Modelling, 60590 Frankfurt, Germany

**Keywords:** anti-platelet factor 4 antibodies, anti-platelet factor 4/heparin antibodies, anti-platelet factor 4/polyanion antibodies, thrombosis, immunothrombosis, antiphospholipid syndrome (APS), heparin-induced thrombocytopenia (HIT), vaccine-induced immune thrombocytopenia and thrombosis (VITT)

## Abstract

Heterogeneous antibodies directed against platelet factor 4 (PF4-Abs) are associated with heparin-induced thrombocytopenia (HIT) and vaccine-induced immune thrombocytopenia and thrombosis (VITT). Emerging evidence suggests that these antibodies may also occur in other thromboembolic diseases. In this pilot study, we assessed the prevalence of PF4-Abs in patients with acute thromboembolism and in patients with thromboembolic antiphospholipid syndrome (APS), a chronic autoimmune thrombophilia. Forty patients with acute thromboembolism and 43 patients with APS were included. Both heparin-dependent and heparin-independent PF4-Abs were evaluated using multiple assay platforms, including a prototype chemiluminescence assay for heparin-independent PF4-Abs, an anti-PF4/polyanion IgG chemiluminescence assay, a latex agglutination assay detecting anti-PF4/polyanion IgG/IgM/IgA antibodies, an anti-PF4/heparin IgG ELISA, and functional platelet activation tests. Across the different assays, positivity in at least one assay was observed in 27.5% (11/40) of patients with acute thromboembolism and 27.9% (12/43) of patients with APS. In most antibody-positive patients, no recent heparin exposure was documented. Functional platelet activation was predominantly absent, arguing against a classical HIT- or VITT-like mechanism. Overall, these results demonstrate assay-dependent PF4-Ab positivity in a substantial proportion of patients with acute and chronic thromboembolic conditions. Their clinical and pathogenic relevance remains unclear and warrants further investigation.

## 1. Introduction

Platelet factor 4 (PF4) is a platelet-derived chemokine that has emerged as a key mediator linking thrombosis, inflammation, and immune activation. PF4 is released from α-granules of activated platelets. Due to its strong positive charge, it interacts with negatively charged molecules such as heparin, glycosaminoglycans, and microbial surfaces [1].

These interactions can alter the structure of PF4 and promote the formation of multimolecular, immunogenic PF4/polyanion complexes. This process can lead to the formation of anti-PF4 antibodies (PF4-Abs), which, under certain circumstances, develop potent platelet-activating properties and trigger immune thrombosis [2].

In addition to its role in blood coagulation, PF4 is also actively involved in the innate immune system. It can bind to bacterial and viral surfaces, facilitate pathogen recognition, and modulate the responses of immune cells, including monocytes and neutrophils [3]. Through the formation of such PF4-containing complexes, PF4 can act as a link between hemostasis and immune activation, thereby promoting antibody generation during inflammatory processes [4]. While physiological PF4-Abs are part of the nonspecific immune defense, pathogenic PF4-Abs can rarely develop. Due to their platelet-activating properties, these antibodies can lead to an increased tendency towards immunothrombosis accompanied by platelet depletion.

**Figure 1:**
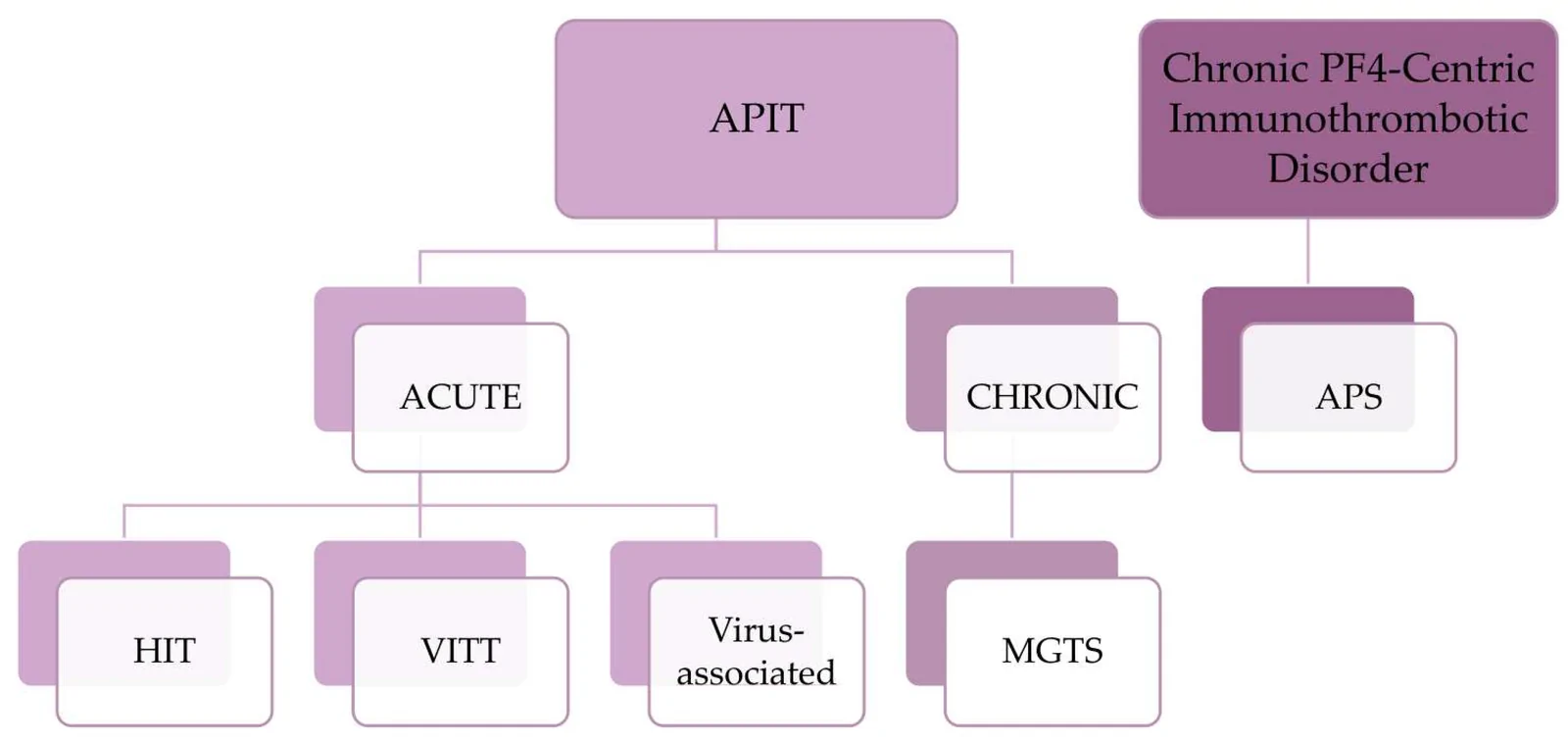
Spectrum of platelet factor 4 (PF4)-associated immunothrombotic disorders. Anti-PF4-associated immune thromboses (APIT) comprise acute disorders, including heparin-induced thrombocytopenia (HIT), vaccine-induced immune thrombocytopenia and thrombosis (VITT), and virus-associated anti-PF4 antibody syndromes, as well as chronic disorders such as monoclonal gammopathy of thrombotic significance (MGTS). In addition, antiphospholipid syndrome (APS) may represent a chronic PF4-centric immunothrombotic disorder in which PF4 contributes to the formation of prothrombotic immune complexes and modulates antiphospholipid antibody-mediated thrombosis [7].

Disorders associated with pathogenic PF4-Abs may be conceptualized as a spectrum of anti-PF4-associated immune thromboses (APIT; see Figure 1). This encompasses acute presentations characterized by a transient trigger and a rapid onset of thrombocytopenia and thrombosis, including heparin-induced thrombocytopenia (HIT [5]), vaccine-induced immune thrombocytopenia and thrombosis (VITT [5]), and virus-induced anti-PF4 syndromes, as well as chronic diseases characterized by persistent pathological antibody production. A prominent example is the monoclonal gammopathy of thrombotic significance (MGTS [6]). Furthermore, other chronic antibody-induced prothrombotic diseases may overlap with the production of PF4-Abs. A potential link has been described for the antiphospholipid syndrome (APS [7]).

Among acute APIT, the role of pathogenic PF4-Abs is best established in HIT and VITT. HIT is a severe immune-mediated side effect of heparin therapy characterized by thrombocytopenia and an increased risk of thrombosis. In HIT, antibodies directed against complexes of PF4 and heparin (PF4/H-Abs) activate platelets via FcγIIa receptors, thereby inducing the strong activation of platelets [8]. A similar mechanism was later identified in VITT, a rare but serious complication associated with SARS-CoV-2 vaccines based on adenovirus vectors. In contrast to HIT, VITT develops without heparin exposure but triggers platelet activation by the formation of pathogenic PF4-antibody immune complexes. This leads to severe thrombocytopenia and thrombosis often at atypical locations [5].

The identification of VITT fundamentally broadened the spectrum of PF4-mediated immune thrombosis beyond classical HIT. There is growing evidence that PF4-Abs can develop in a variety of inflammatory situations, including infections, trauma, surgery, and vaccination [9,10,11,12]. Beyond acute thromboinflammatory syndromes, persistent anti-PF4 immune responses may also contribute to chronic diseases characterized by recurrent thrombotic manifestations. MGTS, a recently recognized prothrombotic disorder in which monoclonal PF4-Abs are produced by a clonal B-cell, may be an example of chronic APIT [6,13]. 

Finally, APS is an acquired autoimmune thrombophilia characterized by persistent autoantibody production and recurrent thrombosis, thereby sharing some clinical and pathogenic features with anti-PF4-mediated diseases. This includes chronic immune activation, thrombocytopenia and dysregulated thromboinflammatory signaling pathways [14]. Recent studies demonstrated that PF4 and its corresponding antibodies interfere with APS pathogenesis through the formation of β2-glycoprotein I (β2-GPI)/PF4 complexes, which enhance the prothrombotic effects of anti-β2-GPI antibodies [15,16]. Therefore, PF4 may represent a common mechanistic link between distinct forms of acute and chronic immunothrombotic disorders. Importantly, the prevalence and clinical relevance of PF4-Abs in APS have not yet been systematically analyzed.

The aim of this study was to investigate the prevalence of various PF4-Abs in patients with acute spontaneous, infection-associated, vaccine-associated, or recurrent thromboembolic events as well as in patients with chronic thromboembolic APS, in order to explore the potential role of PF4-Abs across a broader spectrum of acute and chronic thrombotic disorders. For this purpose, we combined different immunoassays capable of detecting a broad spectrum of PF4 and PF4/H antibodies.

## 2. Results

### 2.1. Patient Characteristics

#### 2.1.1. Group 1: Prospectively Included Patients with Acute Thromboembolism (ATE)

Forty patients with acute thromboembolism (Group 1: ATE) were prospectively included. They were on average 39.5 years old, with a higher proportion of men (65.0%, 26/40). The majority presented with venous thromboembolism (77.5%, 31/40), mainly deep vein thrombosis (DVT) and/or pulmonary embolism (PE). More detailed information is given in Table 1. Patients with acute arterial thromboses (22.5%, 9/40) had experienced ischemic strokes (including transient ischemic attacks (TIAs); 66.7%, 6/9) or myocardial infarction (33.3%, 3/9).

#### 2.1.2. Group 2: Retrospectively Included APS Patients with Chronic Thromboembolic Disease (APS-CTE)

Forty-three APS patients with chronic thromboembolic disease (Group 2: APS-CTE) were retrospectively included. They were on average 46.0 years old, with a higher proportion of women (58.1%, 25/43; see Table 1). The majority of APS patients had developed venous thromboembolism in the past (53.5%, 23/43), while 20.9% had experienced arterial thromboembolism (9/43); 25.6% (11/43) had a history of both venous and arterial thromboembolic events. 81.4% (35/43) had a primary APS, while 18.6% (8/43) had a secondary APS. The majority presented with triple-positive APS (86.0%, 37/43), while five patients had single-positive APS (11.6%). Lupus anticoagulants were detected in all patients; IgG-class antibodies against cardiolipin and β2-glycoprotein I were more common than IgM-class antibodies. All patients except two were anticoagulated at the time of inclusion (95.3%, 41/43). The most commonly used medications were vitamin K antagonists (51.2%, 21/41), followed by direct oral anticoagulants (31.7%; 13/41). The remaining patients received fondaparinux, low-molecular-weight heparin (LMWH), platelet inhibitors (PI), or combinations of coagulation inhibitors.

### 2.2. Vascular Risk Factors

#### 2.2.1. Risk Factors in Patients with Acute Thromboembolism (Group 1: ATE)

Across the entire group (*n* = 40), obesity (Body Mass Index (BMI) > 30 kg/m^2^) was diagnosed in five patients (12.5%).

All patients with venous thromboembolism (*n* = 31) underwent thrombophilia screening, which revealed a heterozygous Factor V Leiden mutation (FVL mutation) in five patients (16.1%). One patient had a homozygous FVL mutation and one additional patient was diagnosed with a heterozygous prothrombin mutation (3.2% each).

In addition, persistently elevated factor VIII activity was detected in eight patients (25.8%). Protein S deficiency was observed in one patient (3.2%). APS was already known in two patients at the time of enrollment in the study and was diagnosed in two additional patients during the course of the study (10.0%).

In one patient, a recurrence of the patient’s tumor (uterine sarcoma) was detected during the course of her treatment, which may have contributed significantly to the development of recurrent venous thrombosis.

The following vascular risk factors were identified in patients with arterial thromboembolism (*n* = 9): Four patients had arterial hypertension, and four additional patients were diagnosed with hypercholesterolemia (44.4% each). One patient also had a history of nicotine abuse (11.1%). During follow-up, a patent foramen ovale (PFO) was detected in two patients (22.2%), and atrial fibrillation was observed in one patient (11.1%).

However, when considering the vascular risk factors of individual patients, their age, and the severity of the thromboembolic event, the presence of these vascular risk factors did not provide a sufficient explanation for the development of arterial thromboembolism in these patients.

#### 2.2.2. Risk Factors in APS Patients with Chronic Thromboembolic Disease (Group 2: APS-CTE)

In the entire group (*n* = 43), 30.2% were obese (BMI > 30 kg/m^2^).

In patients with venous thrombosis (*n* = 34), one patient had a heterozygous prothrombin mutation and one patient presented with a homozygous FVL mutation (2.9% each) in addition to APS.

In total, 70.0% of APS patients with arterial thromboembolism (*n* = 20) had hypercholesterolemia, and 60.0% suffered from arterial hypertension. A PFO was detected in 4 of 20 patients (20.0%), and diabetes mellitus type 2 was present in 15.0%. In addition, four patients had a history of nicotine abuse (20.0%).

### 2.3. Infections & Vaccinations: Group 1: Patients with Acute Thromboembolism (ATE)

The patients who presented with infection-associated thromboembolism (37.5%, 15/40) predominantly suffered from respiratory tract infections (53.3%), presumably viral infections, although the pathogen could generally not be identified. The remaining patients had gastrointestinal and urinary tract infections, and one patient presented with a high temperature only.

Three patients with vaccine-associated thromboembolism were included in the study. In two cases, the vaccinations were booster vaccinations against tetanus, diphtheria, and pertussis, while the third patient received both a hepatitis A and a tick-borne encephalitis vaccination at the same time.

### 2.4. Detection of Different Types of Anti-Platelet Factor 4-Antibodies (PF4-Abs)

#### 2.4.1. Group 1: Patients with Acute Thromboembolism (ATE)

We performed several assays to detect PF4-Abs. Blood samples were collected a median of 30 days (range: 0–111 days) after the thrombotic event had occurred. When considering positivity in at least one of the four assays, antibodies directed against PF4 or PF4-containing complexes were detected in 27.5% (11/40) of patients.

In detail, anti-PF4 IgG antibodies were detected in three patients (7.5%, 3/40) using a prototype chemiluminescence method not yet commercially available (AcuStar anti-PF4 IgG prototype chemiluminescence assay, Werfen), which detects antibodies directed against PF4 alone. Using a commercially available chemiluminescence method, which detects anti-PF4/polyanion IgG antibodies (HemosIL^®^ AcuStar HIT-IgG_(PF4-H)_, Werfen), four patients tested positive (10.0%). Three of these patients were also positive in the anti-PF4 IgG prototype chemiluminescence assay.

Using an ELISA assay (ZYMUTEST^®^ HIA IgG, Hyphen), which detects IgG antibodies directed against complexes of heparin and PF4, positive results were observed in eight patients (20.0%, 8/40).

Using a latex agglutination assay that detects anti-PF4/polyanion IgG, IgM and IgA antibodies (HemosIL^®^ HIT-Ab_(PF4-H)_, Werfen), only one patient tested positive (see Table 2 and Table 3 for more detailed information).

At inclusion, none of the patients showed positive results in the heparin-induced platelet aggregation test (HIPA) and the PF4-induced platelet aggregation test (PIPA). One patient developed transiently positive HIPA results during follow-up (see also Section 2.6). 

#### 2.4.2. Group 2: APS-Patients with Chronic Thromboembolic Disease (APS-CTE)

PF4-Abs were detected in 27.9% (12/43) of patients with chronic APS when positivity in at least one of the four assays was considered. A total of five patients (11.6%, 5/43) tested positive in each of the two chemiluminescence assays, the novel anti-PF4 IgG prototype chemiluminescence assay and the anti-PF4/polyanion IgG chemiluminescence assay, with overlapping positive results in four of five patients. Seven patients (16.3%) tested positive in the ELISA-based anti-PF4/heparin IgG assay. Only one patient tested positive in the latex agglutination-based anti-PF4/polyanion IgG/IgM/IgA assay (see Table 2 and Table 4 for more detailed information).

In addition, one patient had positive results in both platelet activation assays (HIPA and PIPA). Platelet activation was not inhibited by FcγRIIa receptor-blocking antibodies.

### 2.5. Association Between Laboratory Findings and Clinical Presentation

#### 2.5.1. Group 1: Patients with Acute Thromboembolism (ATE)

Across the four assays, 27.5% (11/40) of the patients showed positivity in at least one PF4-Ab assay. Ten of these eleven patients had presented with acute venous thromboses, and only one had developed an arterial thrombosis. This distribution of the types of thrombosis was consistent with the distribution in the entire cohort (see Table 1).

Five of these patients had experienced infection-associated thromboses, another five patients presented with spontaneous thromboses without an identifiable trigger, and one patient had developed a vaccine-associated thrombosis. PF4-Abs were detected in five of the seven patients (71.4%) who had developed infarct pneumonia in the context of a pulmonary embolism. In addition, PF4-Abs were detected in three of four patients who were diagnosed with APS either at inclusion or during the clinical course of the study.

Heparin exposure at the time of the acute thromboembolism was retrospectively analyzed in the entire group. For two patients, no information on a potential heparin exposure was available. 55.3% (21/38) of the residual patients had not received heparin in the last 120 days prior to blood collection. 13.2% (5/38) of the patients had received unfractionated heparin (UFH), while 31.6% (12/38) had been treated with low-molecular-weight heparin (LMWH). Heparin exposure was started at a median of 29 days before blood collection (range: 6–180 days) and lasted for six days (median; range: 1–180 days).

Of the patients who tested positive in at least one of the assays, 54.5% (6/11) had not experienced any heparin exposure, while 36.4% (4/11) had received LMWH within the past 120 days, which had been started 16 days before blood collection (median; range: 13–50 days) and had been continued for five days (median; range: 4–30 days). For one patient who tested positive in the ELISA, no information about previous heparin exposure was available.

#### 2.5.2. Group 2: APS Patients with Chronic Thromboembolic Disease (APS-CTE)

All twelve patients who tested positive in at least one of the assays (27.9%, 12/43) suffered from primary triple-positive APS. However, it should be noted that 69.8% (30/43) of the patients in our entire cohort presented with primary triple-positive APS. A larger proportion of women (66.6%, 8/12) tested positive for PF4-Abs compared to a slightly higher proportion of women in the overall population (58.1%, 25/43). A total of 41.7% (5/12) had a history of venous thromboembolism (primarily DVT and PE) and 41.7% (5/12) a history of arterial thromboembolism (predominantly ischemic stroke), while two patients (16.7%) had developed both types of thrombosis in the past.

In total, 97.7% (42/43) of APS patients had not received heparin in the past 120 days; only one patient received low-molecular-weight heparin (LMWH) for prophylaxis; however, this patient did not test positive for PF4-Abs.

### 2.6. Thrombocytopenia

#### 2.6.1. Thrombocytopenia in Patients with Acute Thromboembolism (ATE)

At baseline, three patients (7.5%, 3/40) presented with thrombocytopenia that could not be explained by any other cause, with platelet counts ranging from 74 to 125 per nanoliter (nL) (cut-off: women < 140/nL, men < 151/nL), but only one of these patients had positive PF4/H-Ab results using the ELISA.

During follow-up, thrombocytopenia developed in two additional patients, occurring twelve and 191 days after enrollment, respectively. Retrospective analysis of the patient who developed thrombocytopenia 191 days after enrollment revealed that thrombocytopenia had already been present at the time of thrombosis but was no longer detectable at study enrollment. In both cases, thrombocytopenia was associated with positive results in the ELISA and the anti-PF4/polyanion IgG chemiluminescence assay. In addition, one of these two patients had weakly positive PF4-Abs in the anti-PF4 IgG prototype chemiluminescence assay; however, the HIPA and PIPA tests were negative.

#### 2.6.2. Thrombocytopenia in APS Patients with Chronic Thromboembolic Disease (APS-CTE)

In the group of APS patients, thrombocytopenia was observed in five patients (11.6%, 5/43), with platelet counts ranging from 48 to 129/nL. PF4-Abs were simultaneously detected in three of these patients, one of whom (patient 3) also showed positive functional test results. Notably, two of these patients had a concomitant diagnosis of immune thrombocytopenia (ITP), and one patient had Evans syndrome.

### 2.7. Association of Clinical Characteristics with Antibody Positivity

We further analyzed potential factors associated with PF4-Ab positivity in at least one of the assays using univariate and multivariate logistic regression analysis. None of the patient characteristics (see Table 1), platelet count, or infection-associated thrombosis in the acute thromboembolism cohort showed a statistically significant association with PF4-Ab positivity.

### 2.8. Follow-Up on Patients with Acute Thromboembolism (Group 1: ATE)

In our prospective cohort of patients with acute thromboembolism, we collected follow-up data on the patients whenever they returned for routine outpatient checkups after having been included in the study. The median follow-up duration was 156 days (range: 13–611 days). No follow-up data were available for six patients, including one patient with a positive anti-PF4/heparin-IgG ELISA result.

All patients who had negative PF4-Ab results at baseline remained negative throughout the complete follow-up period. Among patients with positive results and available follow-up (*n* = 10), two distinct follow-up patterns emerged: 5 of the 10 patients showed a loss of antibody positivity during follow-up. In contrast, in 5 of the 10 patients, persistence of the antibodies at follow-up after approximately 3–20 months was observed. In one patient, a fluctuating course of antibody levels was seen over a total period of 21 months.

In the following, we describe some of these individual patient cases with different clinical courses in more detail.

#### 2.8.1. A Patient with Transient PF4/H-Abs and PF4-Abs (See Figure 2)

This 53-year-old male patient with a heterozygous FVL mutation developed an infection-associated pulmonary embolism accompanied by post-infarction pneumonia. Approximately two weeks earlier, the patient had been suffering from a respiratory tract infection characterized by fatigue, fever, and cough. Four days before the pulmonary embolism was detected, pneumonia was diagnosed. At the same time, the patient had mild thrombocytopenia with a platelet count of 121/nL (cut-off < 151/nL). He received LMWH for 5 days starting from the time of diagnosis, after which he was switched to rivaroxaban.

**Figure 2:**
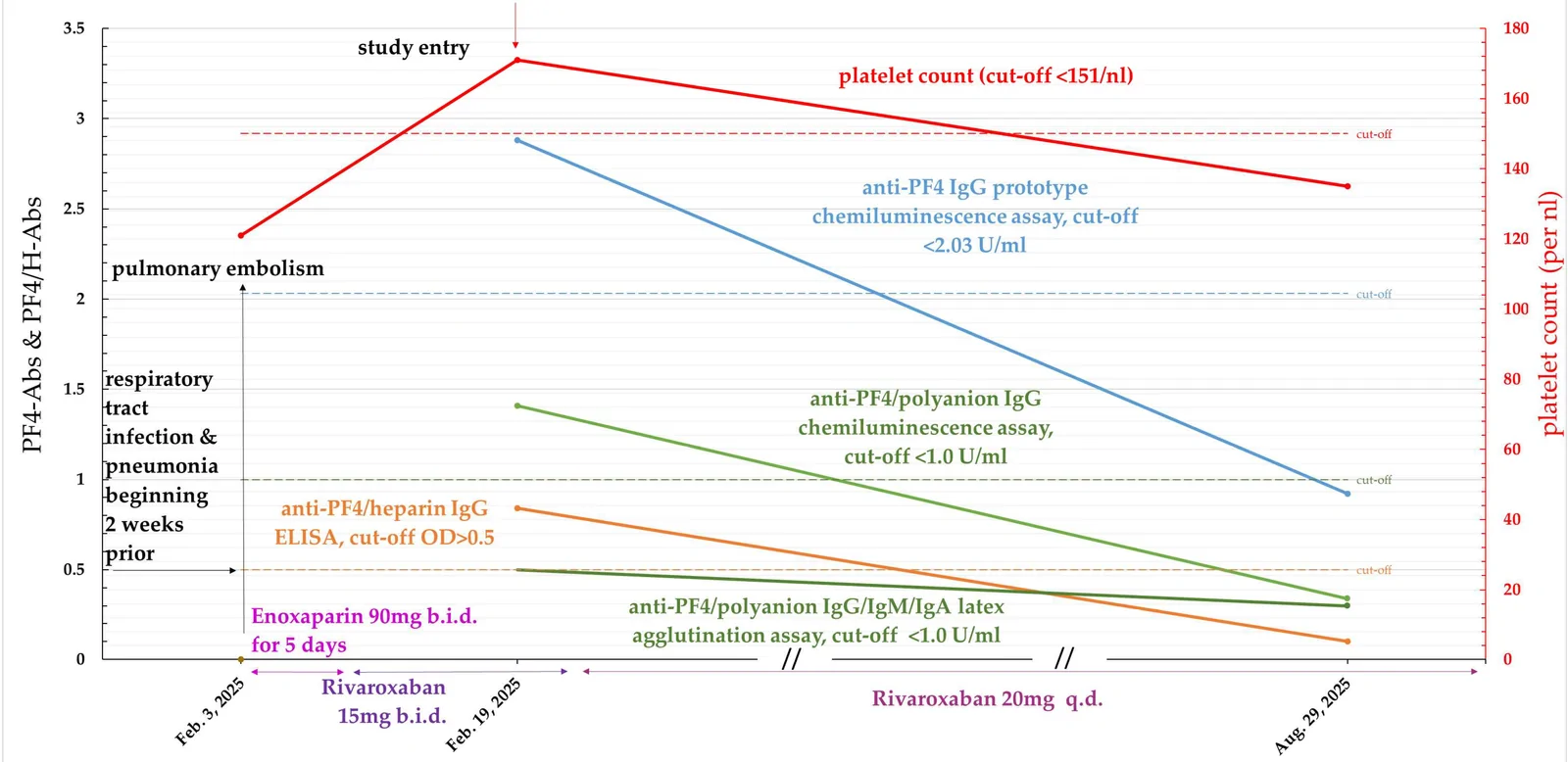
Group 1: Patient no. 18 with transient PF4/H-Abs and PF4-Abs. q.i.: once daily, b.i.d.: twice daily, U/mL: units per milliliter, OD: optical density, nL: nanoliter.

The blood sample was collected at our center 16 days after PE was diagnosed. Thrombocytopenia was no longer present at that time (platelet count = 172/nL). Heparin-independent PF4-Abs using the new anti-PF4 IgG prototype chemiluminescence assay, as well as anti-PF4/heparin-IgG antibodies using the ELISA and anti-PF4/polyanion IgG antibodies using the anti-PF4/polyanion IgG chemiluminescence assay, were detected. Functional tests were negative.

At follow-up approximately 6 months later, no PF4-Abs were detectable; nevertheless, mild thrombocytopenia was again observed with a platelet count of 135/nL.

#### 2.8.2. A Patient with Persistent PF4/H-Abs and Transient PF4-Abs Who Developed APS (See Figure 3)

This 38-year-old male patient also suffered from an infection-associated pulmonary embolism with concomitant post-infarction pneumonia. He had been complaining of a recurrent upper respiratory tract infection for about two months. He did not receive any heparin.

In the blood sample taken at baseline, obtained eight days after the diagnosis of pulmonary embolism, positive results were obtained in both chemiluminescence assays, indicating the presence of heparin-independent anti-PF4 IgG antibodies and anti-PF4/polyanion IgG antibodies. Follow-up data were available for up to 15 months after the thrombotic event. After approximately ten months, the patient no longer tested positive in the prototype chemiluminescence assay; however, he remained positive in the anti-PF4/polyanion IgG chemiluminescence assay even after 15 months.

In addition, during follow-up, based on the persistent presence of positive lupus anticoagulants, a primary APS, which had not been previously identified, was detected.

**Figure 3:**
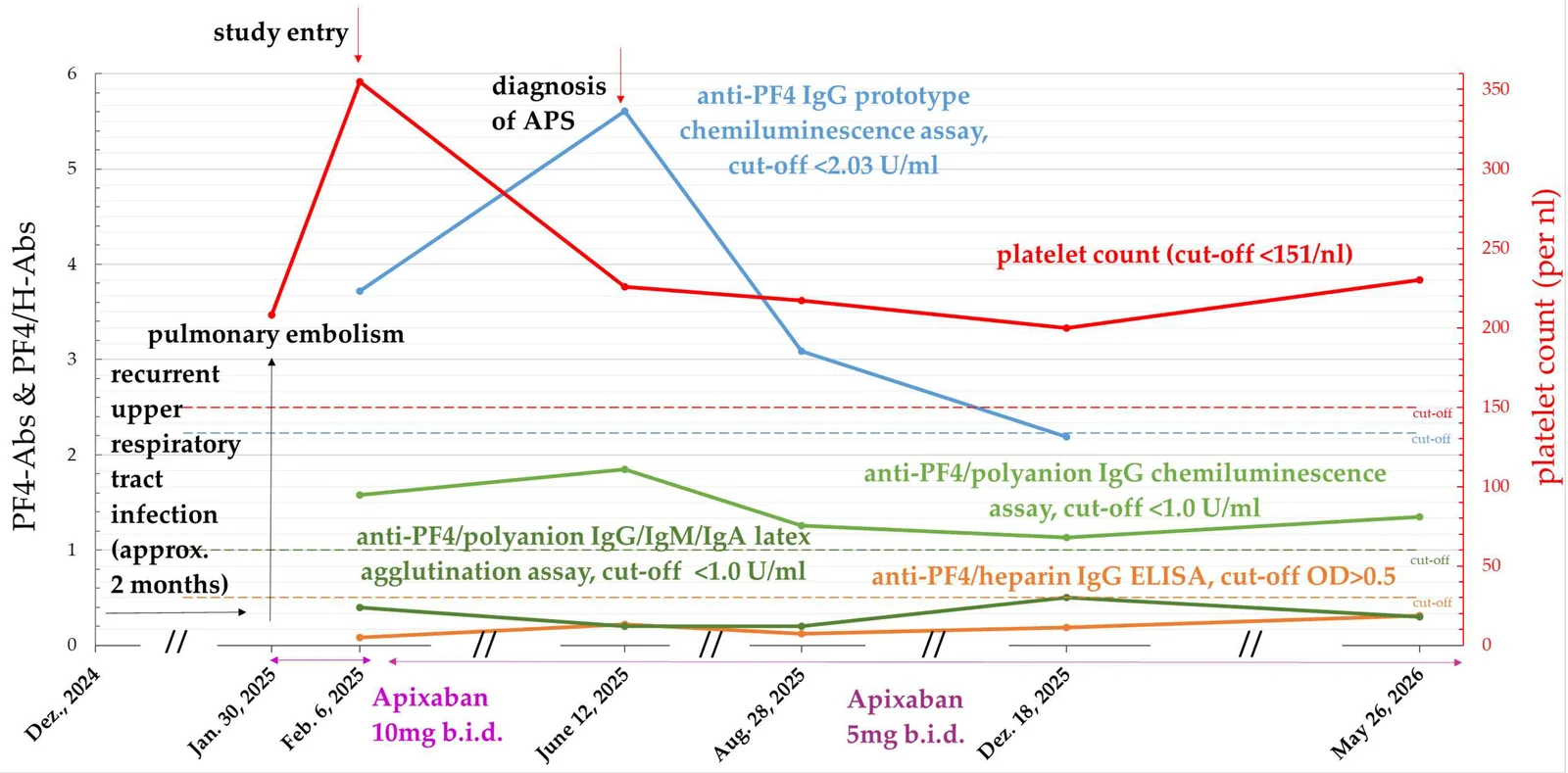
Group 1: Patient no. 17 with persistent PF4/H-Abs and transient PF4-Abs who developed APS. q.i.: once daily, b.i.d.: twice daily, U/mL: units per milliliter, OD: optical density, nL: nanoliter.

#### 2.8.3. A Patient with Known APS, Recurrent Thromboses Despite Anticoagulation and Transient PF4/H-Abs (See Figure 4)

A 33-year-old woman with known triple-positive APS developed a vaccine-associated recurrence of pelvic vein thrombosis despite being on therapeutic anticoagulation with phenprocoumon. The patient had received a booster vaccination against tetanus, diphtheria, and pertussis (Tdap). Thirteen days after the vaccination, she noticed swelling of her left leg during physical exercise; 24 days after the vaccination, she was diagnosed with recurrent thrombosis of the left pelvic vein. She was then treated with enoxaparin for 4 days in addition to phenprocoumon. The patient had a history of two previous episodes of deep vein thrombosis and a TIA, requiring treatment with phenprocoumon, which she had been taking regularly.

Although the phenprocoumon dose was subsequently increased and therapeutic anticoagulation with fondaparinux was initiated, the thrombosis continued to progress and D-dimer levels remained elevated, accompanied by severe pain. In addition to the highly elevated levels of APS antibodies, the patient continued to show weakly positive results in the anti-PF4/heparin IgG ELISA, and, for the first time, anti-PF4/polyanion IgG antibodies were detected using the anti-PF4/polyanion IgG chemiluminescence method; however, thrombocytopenia was not present and functional tests were negative at that time.

The patient subsequently underwent six plasma exchanges, after which the PF4-Abs were no longer detectable. Nevertheless, the patient developed mild thrombocytopenia (platelet counts between 105 and 114/nL, cutoff < 140/nL). IgG antibodies directed against glycoprotein complexes IbIX, IIbIIIa, and GPV on the patient’s platelets were additionally detected.

During plasmapheresis, the patient was anticoagulated with argatroban; later on, she received fondaparinux, and phenprocoumon was discontinued.

**Figure 4:**
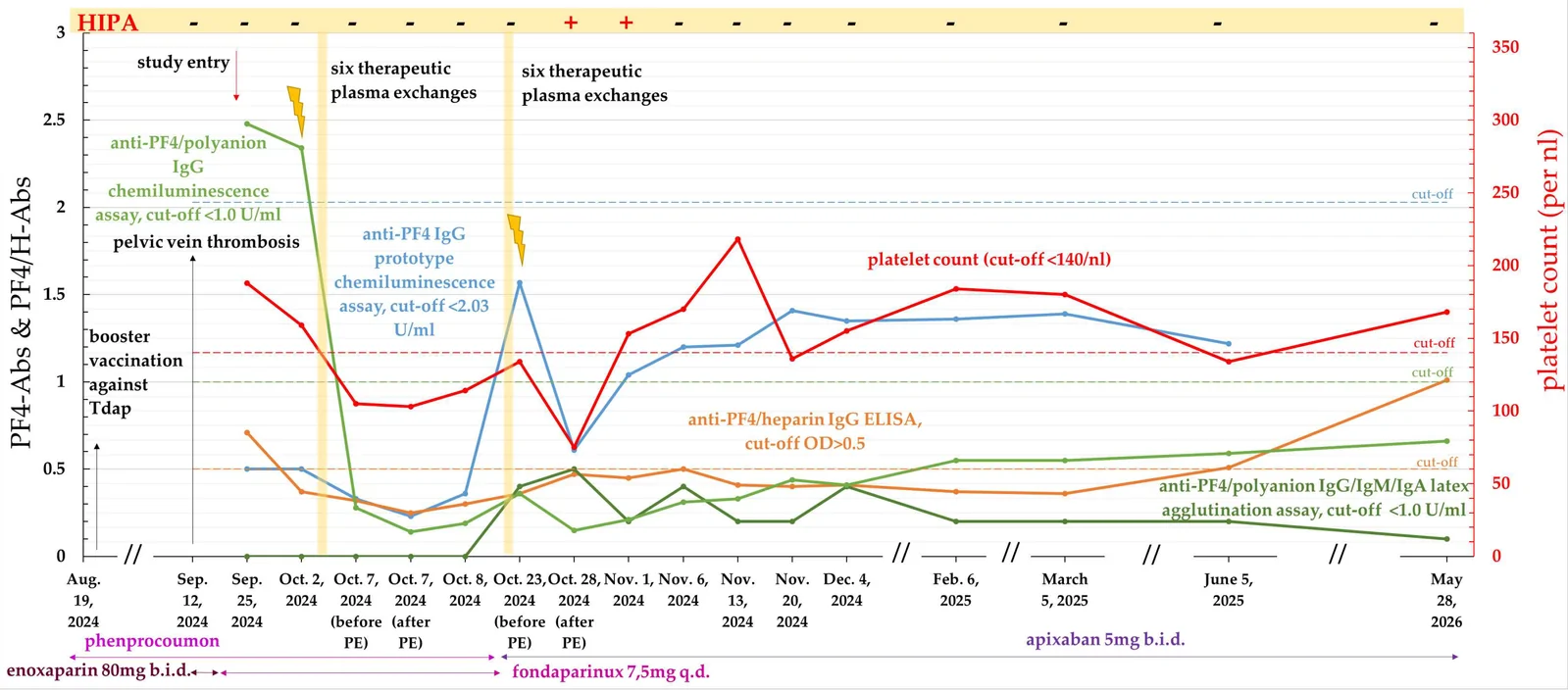
Group 1: Patient no. 9 with known APS, recurrent thromboses despite anticoagulation and transient PF4/H-Abs. Recurrent thromboses are marked by lightning bolts. q.i.: once daily, b.i.d.: twice daily, U/mL: units per milliliter, OD: optical density, nL: nanoliter.

The patient experienced a recurrence of symptoms 14 days after completion of plasmapheresis. Subsequent ultrasound examination revealed recurrent pelvic vein thrombosis, while laboratory testing again demonstrated elevated D-dimer levels, mild thrombocytopenia, and a weakly positive anti-PF4/heparin ELISA result, prompting the patient to undergo plasmapheresis again. In addition, treatment with rituximab was initiated.

Following plasmapheresis, the patient was switched to anticoagulation with apixaban. During plasmapheresis, PF4-Abs were closely monitored: despite plasma exchange, she continued to show borderline results in the ELISA assay and, for the first time, a positive HIPA assay result (see Figure 4). Likewise, progressive thrombocytopenia developed despite plasmapheresis, with platelet counts dropping to 75/nL. Nine days after the first positive HIPA result, the test result returned to negative.

Throughout the patient’s subsequent follow-up (one and a half years since these recurrent thrombotic events), the patient has shown persistently borderline results on the PF4/H ELISA (OD > 0.3 and <0.5); the other PF4-Ab assays remained negative.

Most recently, the patient showed a new increase in anti-PF4/heparin-IgG antibodies using the ELISA method, however, without the corresponding clinical symptoms, and platelet counts have remained in the lower normal range.

## 3. Discussion

The primary objective of this study was to determine the prevalence of PF4-Abs in prospectively included patients with spontaneous, infection- or vaccine-associated or recurrent acute thromboembolism, as well as in retrospectively included patients with known thromboembolic APS as an example of a chronic thromboembolic disorder.

A key finding was that PF4-Abs are also detectable outside of classic contexts such as HIT and VITT, although detection rates vary among the individual assays. While the anti-PF4/heparin IgG ELISA yielded the highest positivity rates, with 20.0% of patients with acute thromboembolism and 16.3% of APS patients testing positive, the anti-PF4/polyanion IgG chemiluminescence assay identified PF4-Abs in 10.0% and 11.6% of these patients, respectively. Importantly, the anti-PF4 IgG prototype chemiluminescence assay also detected heparin-independent anti-PF4 IgG antibodies in 7.5% of patients with acute thromboembolism and in 11.6% of APS patients.

### 3.1. Detection of PF4-Abs in Acute Thromboembolism

In addition to their established role in HIT and VITT, the presence of PF4-Abs has also been described in different clinical situations. For example, Hursting et al. reported a prevalence of these antibodies in approximately 4.3–6.6% healthy blood donors using the PF4 Enhanced^®^ PF4/polyvinyl sulfonate ELISA (Genetics Technology Institute, GTI), a polyspecific assay detecting IgG, IgA, and IgM antibodies against PF4/polyanion complexes [17].

Similarly, Hursting and Arepally detected PF4-Abs in about 1.0–4.3% of healthy individuals. In their literature analysis, antibody detection was performed using different commercial immunoassays, including the Asserachrom^®^ HPIA PF4/heparin ELISA (Diagnostica Stago), the PF4 Enhanced^®^ PF4/polyvinyl sulfonate ELISA (GTI), and the DiaMed particle gel immunoassay (PGIA) [18].

Furthermore, Selling et al. reported even higher prevalences in critically ill patients receiving intravenous UFH: approximately 17.2% of patients in the intensive care unit tested positive for anti-PF4/heparin antibodies, using in-house anti-PF4/heparin ELISAs for IgG, IgM, and IgA antibodies, with a cutoff of 0.5 OD, although only a very small proportion developed clinically manifest HIT [19].

However, it is noteworthy that all intensive care patients had been treated with intravenous UFH, whereas no patient in our cohort had received UFH. Only a small proportion of our patients had received LMWH, whereas for many, no heparin administration was documented. This difference regarding heparin exposure should be taken into account when comparing the study results.

Given this background, the detection of antibodies directed against PF4 or PF4-containing complexes in our cohort seems heterogeneous, with detection rates appearing higher in most assays (anti-PF4/polyanion IgG chemiluminescence assay, anti-PF4/heparin IgG ELISA, and anti-PF4 IgG prototype chemiluminescence assay) than the rates reported in the general population and even among heparin treated intensive care patients. However, detection rates varied substantially depending on the assay employed. Comparisons across studies should therefore be interpreted with caution, taking into account differences in both the study populations and the assay methodologies used.

### 3.2. Detection of PF4-Abs in Patients with Positive Antiphospholipid Antibodies or APS-Associated Chronic Thromboembolic Disease

There are only a few studies to date on the prevalence of PF4-Abs in patients with either positive antiphospholipid antibodies or APS, and the results vary considerably. While Martin-Toutain et al. found anti-PF4/heparin antibodies in 11% of patients with antiphospholipid antibodies (aPL), with or without associated APS using the Asserachrom^®^ HPIA PF4/heparin ELISA (Diagnostica Stago) [20], Martinuzzo et al. detected anti-PF4/heparin antibodies in 21.2% of patients with antiphospholipid antibodies [21] using the same assay.

However, PF4 may be involved in APS pathophysiology, particularly through its interaction with β2-GPI. Together with PF4, β2-GPI forms larger antigenic complexes that are more easily recognized by APS antibodies than β2-GPI alone. Villani et al. detected antibodies against the β2-GPI/PF4 complexes in 34.2% of patients with thrombotic APS; a positive result was associated with an increased incidence of venous thrombotic events and a triple-positive APS.

Nevertheless, it remains unclear whether the PF4-Abs detected in our APS cohort contribute to thrombotic manifestations or merely reflect persistent immune activation. The detection of PF4-Abs across multiple assays suggests that these antibodies may represent an additional immunological feature in a subgroup of APS patients. However, further studies are needed to determine whether these antibodies possess functional prothrombotic properties and contribute to the clinical manifestations of APS.

### 3.3. Potential Mechanisms Underlying PF4-Ab Generation

The mechanisms underlying the generation of PF4-Abs in these patient groups have not yet been fully elucidated. Several hypotheses may account for the unexpectedly high assay-dependent prevalence observed in our two cohorts.

First, PF4-Abs may arise as part of an immune response to external triggers such as infections or vaccination. This is consistent with the known ability of PF4 to bind to negatively charged bacterial and viral surfaces, leading to the formation of immunogenic complexes [3].

In this context, PF4 and PF4-Abs may be part of an evolutionarily ancient antimicrobial defense mechanism that links innate and adaptive immunity. At the same time, PF4-Abs may, under certain circumstances, contribute to thromboinflammatory processes through their procoagulant and proinflammatory effects [1].

Second, PF4-Abs might be linked to systemic inflammation. Proinflammatory states are associated with enhanced platelet activation and PF4 release, which may facilitate the formation of immunogenic PF4-containing complexes. Observational studies in critically ill patients, such as those in intensive care or patients with COVID-19 infections and severe disease, have reported PF4-Abs in the absence of clear clinical features of HIT, leading some authors to interpret these antibodies as markers of generalized immune activation rather than evidence of a direct pathogenic mechanism [19,22].

In addition, thromboembolic events are typically multifactorial in origin, making it inherently difficult to delineate the relative contribution of individual triggers, including PF4-Abs.

Moreover, these two scenarios are not mutually exclusive and may coexist within the same patient population. It is conceivable that some of the PF4-Abs have pathogenic potential, while others merely reflect underlying immune activation. However, based on the available data, it is not possible to determine the clinical relevance of the different types of PF4-Abs so far.

### 3.4. Functional Characterization of Detected PF4-Abs

Despite the frequent detection of PF4-Abs observed in both cohorts, there was only limited evidence of platelet-activating PF4-Abs. None of the patients with acute thromboembolism tested positive in either the HIPA or the PIPA at baseline, whereas only a single APS patient showed transiently positive results in both assays.

Based on these results, routine functional testing of all PF4-Ab-positive patients does not appear to be warranted. However, in patients with new-onset or worsening thrombocytopenia, particularly in association with thrombosis, further functional testing may be indicated. The clinical relevance of PF4 antibodies detected in the absence of thrombocytopenia remains to be clarified.

At the same time, the absence of HIPA or PIPA reactivity should not be interpreted as definitive evidence against the biological activity of the detected antibodies. Both assays are designed to identify platelet-activating antibodies and therefore assess only a specific aspect of PF4-Ab function.

Recent studies of platelet-activating anti-PF4 disorders have demonstrated that pathogenic anti-PF4 immune complexes may induce thromboinflammatory responses not only through platelet activation but also through activation of monocytes, neutrophils, and endothelial cells, resulting in tissue-factor expression, release of neutrophil extracellular traps, release of von Willebrand factor, and enhanced thrombin generation. Thus, PF4-Abs may potentially contribute to thrombosis through multiple interconnected cellular pathways beyond those captured by conventional platelet activation assays [23].

Further studies are required to determine whether the antibodies detected in these patient groups with acute or chronic thromboembolic disease possess additional clinically relevant prothrombotic properties or merely represent markers of underlying immune activation.

### 3.5. Assay-Dependent Variability and Antibody Heterogeneity

A notable finding was the limited concordance between the different assay systems. While a partial overlap was observed between the commercially available chemiluminescence assay and the prototype assay, the latex agglutination assay yielded almost no positive results. In contrast, the ELISA detected a substantial number of positive samples, many of which did not show consistent overlap with any of the other test systems.

This discrepancy may reflect the increasingly recognized heterogeneity of PF4-Abs [5]. Different assays may not detect all PF4-Abs with the same sensitivity, particularly if the antibodies recognize different epitopes or binding sites on PF4 that are not equally represented in all test systems [24]. Supporting this concept, Dabbiru et al. reported on patients with a VITT-like clinical presentation who tested positive in both chemiluminescence assays, suggesting either cross-reactivity of the antibodies due to overlapping epitopes on PF4 or the coexistence of distinct PF4-Ab populations [25]. Consequently, some subpopulations of PF4-Abs may remain undetected depending on the test used, resulting in an incomplete representation of the entire antibody repertoire.

The combined use of different assays may capture a broader spectrum of PF4-antibodies, as individual assays may detect distinct antibody populations depending on their antigenic target and assay configuration.

Recent findings further support this concept by demonstrating platelet-activating anti-PF4 antibodies in HIT that were not detected by conventional PF4/heparin ELISAs, providing further evidence for distinct PF4-Ab specificities that may not all be captured by existing assay platforms [26,27].

### 3.6. Antibody Persistence

Further evidence supporting the heterogeneity of PF4-Abs was provided by the marked variability in antibody persistence observed during follow-up. While PF4-Abs became undetectable in some patients within weeks to months, others remained antibody-positive for up to six months or even longer. This pattern appears to span a broad range between the relatively transient antibody response typically observed in HIT [28] and the more prolonged persistence reported, for example, in VITT [29].

The observed variability in antibody persistence further supports the concept that PF4-Abs represent a heterogeneous group of antibodies with potentially distinct biological properties.

### 3.7. Limitations and Future Research Implications

First, the two study cohorts differed in their study design. While the cohort with acute thromboembolism was recruited prospectively and followed longitudinally, the analysis of the APS cohort was retrospective, and no follow-up samples were available. In addition, follow-up duration was not uniform across all prospectively included patients, which may have affected estimates of antibody persistence. Furthermore, both cohorts were relatively small, limiting statistical power and the generalizability of the findings.

Second, no control group of healthy subjects or patients with infections was included and analyzed simultaneously. Therefore, the present study does not allow for a direct comparison of PF4 antibody detection between the cohorts studied and other populations. Although most test-specific positivity thresholds were established by the respective manufacturers through testing of independent reference populations, these data cannot replace a control cohort analyzed concurrently, as test platforms, study populations, and clinical contexts differ. Comparisons with previously reported prevalences in healthy individuals or other patient groups should therefore be interpreted with caution.

Third, patient recruitment for the cohort with acute thromboembolism was based on the clinical assessment of the treating physicians, which may have led to selection bias.

Finally, given the absence of samples obtained prior to the thromboembolic event, it remains unclear whether the detected PF4-Abs preceded the thrombotic event or developed only subsequently.

Despite these limitations, the findings raise important questions for future research. Further studies are needed to clarify the biological and clinical relevance of PF4-Abs detected outside the context of classical HIT and VITT. In particular, it remains to be determined whether these antibodies exert biological effects through thromboinflammatory mechanisms beyond platelet activation or merely represent markers of underlying unspecific immune activation.

Longitudinal studies with larger patient cohorts and standardized follow-up are needed to better characterize antibody persistence and identify factors associated with transient versus persistent antibody responses and their clinical significance.

Furthermore, the variability observed between different assay systems underscores the need for further investigation into assay-specific differences in PF4-Abs. Finally, the observation that PF4-Abs appeared to occur more frequently in patients with infection-associated and recurrent thromboembolic events warrants further investigation and may provide additional insights into the mechanisms underlying PF4-Ab formation and persistence.

## 4. Materials and Methods

### 4.1. Prospective Inclusion of Patients with Acute Thromboembolism (Group 1: ATE)

This observational monocentric pilot study aimed to determine the prevalence of antibodies directed against different epitopes of PF4 and was conducted in the Department of Hemostaseology at the Cardioangiological Center of the Bethanien Hospital (CCB) in Frankfurt am Main, Germany.

After receiving ethical approval on 22 March 2024 (approval number: 2023-3513-evBO), patient recruitment was started on 1 June 2024, and ended on 31 December 2025. The study was registered at Clinicaltrials.gov (ID: NCT06650826). When patient recruitment was started the prototype anti-PF4 assay was not yet available in our laboratory. These assays were provided by the company (Werfen) after the first patients had already been included in the study.

**Figure 5:**
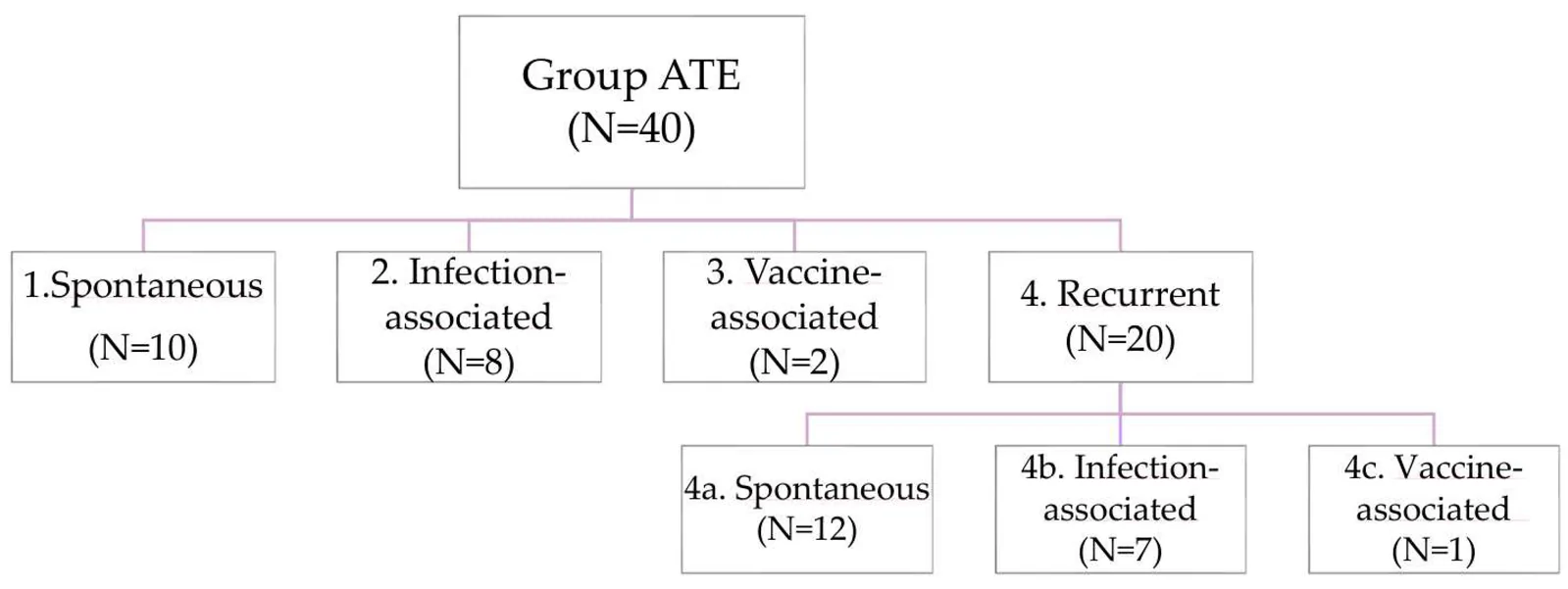
Overview of the ATE group: patients with acute venous and/or arterial thromboembolism. The group can be divided into four subgroups based on their etiology: spontaneous, infection- or vaccine-associated, and recurrent thrombosis.

We prospectively recruited consecutive patients with acute thromboembolism who presented to our vascular outpatient clinic (see Figure 5).

Patients were included when they met the following inclusion criteria:

• Age 18–60 years;
• Venous and/or arterial thrombosis within the last 120 days;
• Thromboembolic event following vaccinations or infections within 30 days prior to the onset of the thrombotic event; or
• Spontaneous thromboses of unknown origin; or
• Recurrent thromboses despite anticoagulant therapy.

Exclusion criteria were:

• Risk-associated venous thromboses due to immobilization, surgery, casting, fractures, or sprains;
• Risk-associated arterial thromboses, such as those with multiple vascular risk factors, including hypercholesterolemia, diabetes mellitus, arterial hypertension, or nicotine abuse.

Patients who participated in the prospective observational study were required to sign a written informed consent before the start of the examinations.

### 4.2. Retrospective Inclusion of Patients with Antiphospholipid Syndrome (APS) with Chronic Thromboembolic Disease (Group 2: APS-CTE)

In addition, we decided to retrospectively include retained plasma samples of APS patients with chronic thromboembolic disease, who visited our outpatient clinic on a regular basis between 1 June 2024, and 31 December 2025.

**Figure 6:**
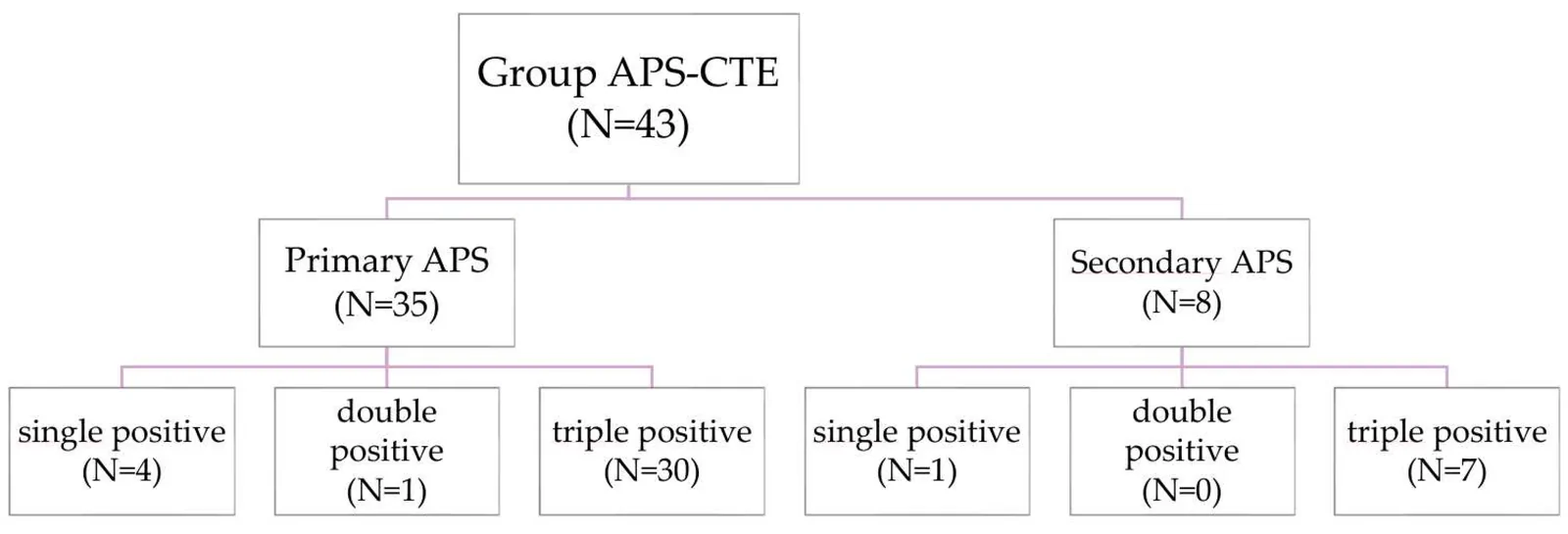
Group APS-CTE: patients with chronic thromboembolic events in the context of an antiphospholipid syndrome (APS).

Patients were only included when they fulfilled the scientific definition of an APS according to the current guidelines of the American College of Rheumatology (ACR) and the European Alliance of Associations for Rheumatology (EULAR) [30] (for more details see Figure 6).

Inclusion criteria:

• Written consent for the use of residual blood samples for research purposes;
• Clinical criteria: venous and/or arterial thromboembolism;
• Laboratory criteria: a persistently positive antiphospholipid antibody (aPL) test within three years of the clinical criterion;
  ○ A lupus anticoagulant test;
  ○ and/or
  ○ Moderate-to-high titers of anticardiolipin (IgG or IgM);
  ○ or
  ○ Moderate-to-high titers of anti-β_2_-glycoprotein-I antibodies (IgG or IgM).

Exclusion criterion:

• APS patients with exclusively obstetric complications.

### 4.3. Patient Data Collection and PF4-Ab Assays

Patients of Group 1 gave written informed consent to an extended blood draw as part of their routine examination, while patients of Group 2 had agreed to the use of their residual blood samples obtained from routine examinations for scientific purposes with written consent in the past.

Clinical data such as gender, age, comorbidities, cardiovascular risk factors, medical history, and medication were retrieved from each patient’s electronic medical record.

The following blood parameters were measured in all patients: complete blood count, routine coagulation parameters including prothrombin time (PT), activated partial thromboplastin time (aPTT), fibrinogen, D-dimer, transaminases, and creatinine.

Patients with venous thrombosis underwent thrombophilia screening, including antithrombin activity, protein C- and protein S-activity, free protein S antigen, factor VIII activity, genetic testing for a FVL mutation or prothrombin mutation, detection of lupus anticoagulants and APS antibodies (see below), as well as levels of homocysteine, and von Willebrand factor antigen.

In patients with arterial thrombosis, vascular risk factors were assessed, including lipid levels (low-density lipoprotein (LDL), high-density lipoprotein (HDL), triglycerides, cholesterol, lipoprotein a and HbA1c). In addition, the presence of arterial hypertension, nicotine abuse, a patent foramen ovale, and atrial fibrillation was documented.

Furthermore, various PF4-Ab assays were conducted in all patients: The anti-PF4/heparin IgG ELISA assay, which is based on PF4/heparin complexes (ZYMUTEST^®^ HIA IgG, HYPHEN BioMed, Neuville-sur-Oise, France, [31]), the heparin-induced platelet activation assay (HIPA, [10]), and the PF4-induced platelet activation assay (PIPA, [10]) were performed at the Institute of Transfusion Medicine at the University Hospital Greifswald, Germany.

In addition, the anti-PF4/polyanion IgG/IgM/IgA latex agglutination assay (HemosIL^®^ HIT-Ab(PF4-H), ACL TOP, Werfen, Barcelona, Spain) and the commercially available anti-PF4/polyanion IgG chemiluminescence assay (HemosIL^®^ AcuStar HIT-IgG(PF4-H), Werfen, Barcelona, Spain, [32]) were performed at the coagulation laboratory of the CardioAngiology Center Bethanien Hospital (CCB) in Frankfurt, Germany. Both assays are based on PF4/polyvinyl sulfonate (PF4/PVS) complexes rather than PF4/heparin complexes.

**Figure 7:**
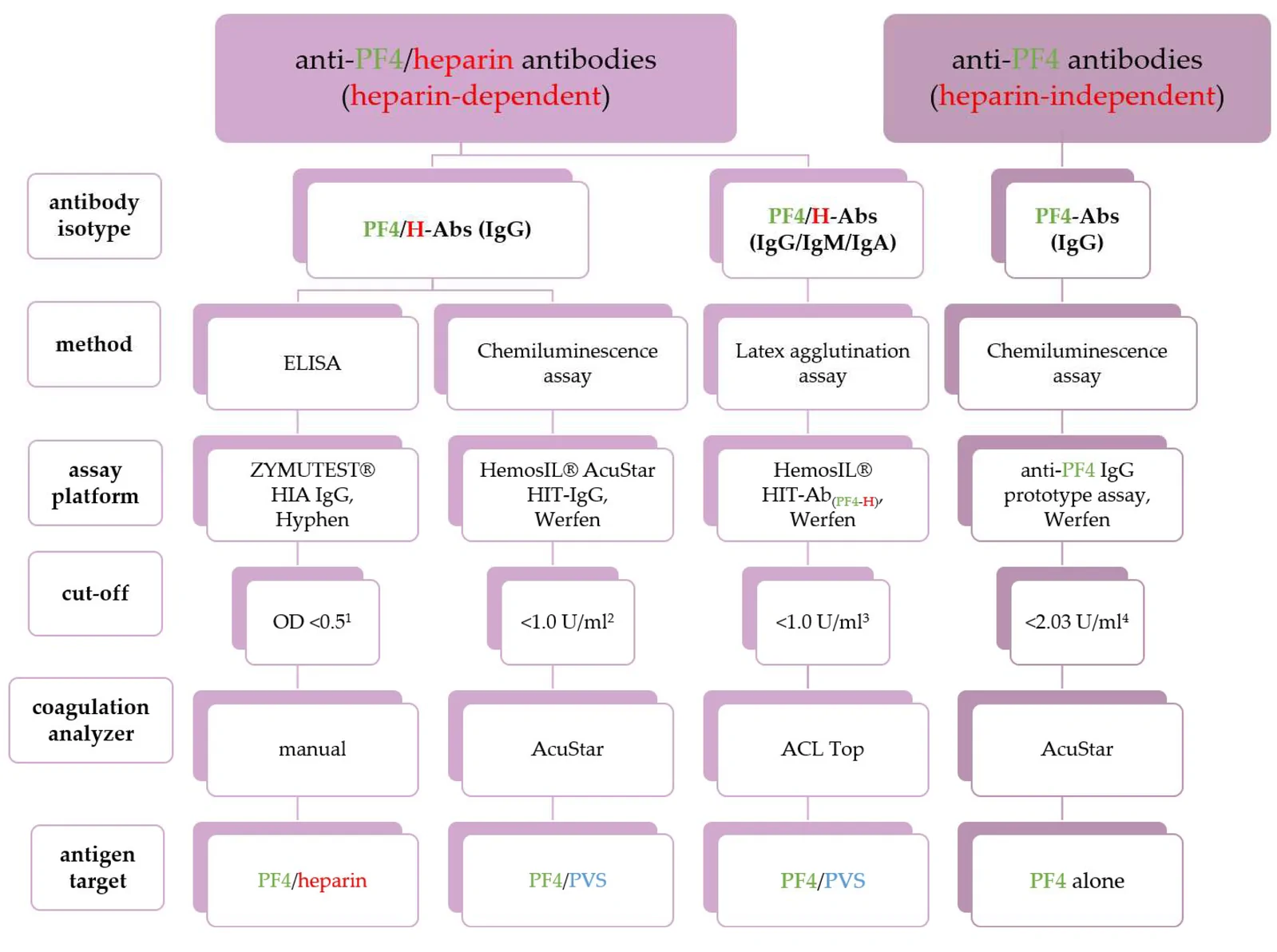
Overview of the various PF4-Ab assays performed in the study, illustrating differences in antibody isotypes, detection methods, assay platforms, coagulation analyzer, and antigen targets. OD: optical density, PF4-Abs: anti-PF4 antibodies (heparin-independent), PF4/H-Abs: anti-PF4/heparin antibodies, PVS: polyvinyl sulfonate. ^1^ the manufacturer’s reference range was established based on a study of 149 healthy adults; the central 90% of the results ranged from 0.02 to 0.19 OD [36]. In accordance with the manufacturer’s instructions, a threshold value of >0.5 OD was used in this study to define a positive result. Samples with OD values between 0.3 and 0.5, which the manufacturer classifies as weakly positive, were classified as negative. ^2^ the cut-off value was established through a ROC analysis using 87 samples from patients with suspected HIT, including 45 SRA-positive and 42 SRA-negative samples [37]. ^3^ the reference interval was determined using samples from 131 healthy donors, yielding a 95% reference interval of 0.0–0.7 U/mL, as well as using an additional cohort of 122 patients with suspected HIT, which yielded a 95% reference interval of 0.0–0.9 U/mL [38]. ^4^ the cut-off value was defined at the 99th percentile of 199 healthy serum and plasma donors (Werfen, Anti-PF4 IgG prototype assay, unpublished results, 2026).

Additionally, the presence of heparin-independent PF4-Abs was determined by a new prototype chemiluminescence assay (anti-PF4 IgG prototype assay, AcuStar, Werfen, Barcelona, Spain), which only detects antibodies directed against PF4 not complexed to heparin. As previously described [33], the assay is based on magnetic beads, on which PF4 alone is immobilized. The cutoff for the anti-PF4 IgG prototype chemiluminescence assay was established by testing VITT and control samples [34,35]. Cross-reactivity of the anti-PF4 IgG prototype chemiluminescence assay with anti-cardiolipin and anti-β2-glycoprotein I antibodies had been ruled out by the manufacturer of the assay (for more details see Figure 7).

Antiphospholipid antibodies were measured in all patients using the following assays: Anticardiolipin IgG and IgM antibodies and anti-ß2 glycoprotein I IgG and IgM antibodies were analyzed using an automated chemiluminescence immunoassay (HemosIL^®^ AcuStar Anti-Cardiolipin IgG and IgM; HemosIL^®^ AcuStar Anti-β2-Glycoprotein-I IgG and IgM, Werfen, Barcelona, Spain).

Lupus anticoagulants were detected using the dilute Russell’s Viper Venom Time Assay (HemosIL^®^ dRVVT Screen and HemosIL^®^ dRVVT Confirm, Werfen, Barcelona, Spain). Additionally, we used a lupus-sensitive aPTT-based assay (MIXCON LA-test, Werfen, Barcelona, Spain) to detect lupus anticoagulants.

### 4.4. Statistical Analysis

The primary objective was to determine the prevalence of various PF4-Abs in both acute and chronic thromboembolic diseases.

The data were analyzed descriptively. Continuous variables are presented as medians and ranges. Categorical variables are presented as frequencies and percentages.

In addition, univariate and multivariate logistic regression analyses were performed to explore potential factors associated with PF4-Ab positivity. The multivariate analysis was performed with backward variable selection using a significance level of α = 0.05. Due to the descriptive nature of the study, no further inferential statistical analyses were performed. Regression analysis was performed with R version 4.4.2 (R Foundation for Statistical Computing, Vienna, Austria).

## 5. Conclusions

In this pilot study, PF4-Abs were frequently detected in patients with thromboembolic events distinct from classical HIT and VITT. Notably, antibody detection was strongly assay-dependent, suggesting substantial heterogeneity and indicating that current diagnostic approaches capture only a fraction of the underlying antibody spectrum.

The observed variability in antibody persistence further supports the hypothesis of heterogeneous antibodies and indicates that both transient and sustained anti-PF4 responses may occur, although this requires confirmation. Importantly, no platelet activation was detected in functional assays, arguing against a mechanism resembling classical anti-PF4-mediated disorders. Nevertheless, a potential pathogenic role cannot be excluded, as mechanisms not captured by the applied functional assays may be involved.

Overall, our results show that antibodies directed against PF4 and PF4-containing complexes can be detected across different acute and chronic thromboembolic conditions, but their clinical and pathogenic relevance remains unclear. Given significant test-dependent differences and marked variability in antibody persistence, PF4-Abs appear to be a heterogeneous immunological phenomenon, underscoring the need for a more differentiated clinical interpretation and further investigation of their clinical relevance beyond the classic contexts of HIT and VITT.

## Figures and Tables

**Table 1:** Patient characteristics. Group 1: patients with acute thromboembolism (ATE, *n* = 40); Group 2: APS patients with chronic thromboembolic disease (APS-CTE, *n* = 43).

	Group 1: Patients with Acute Thromboembolism (ATE)	Group 2: APS Patients with Chronic Thromboembolic Disease (APS-CTE)
***Number of patients (n)***	40	43
*Female % (counts/n)*	35.0% (14/40)	58.1% (25/43)
*Age (years) median [range]*	39.5 [18–59]	46.0 [20–78]
*BMI (kg/m^2^) median [range]*	25.3 [18.3–34.2]	27.2 [18.7–40.9]
***Thromboembolism***		
***VT % (counts/n)***	77.5% (31/40)	53.5% (23/43)
*DVT % (counts/n)*	38.7% (12/31)	43.5% (10/23)
*PE % (counts/n)*	25.8% (8/31)	8.7% (2/23)
*DVT and PE % (counts/n)*	16.1% (5/31)	39.1% (9/23)
*Other* *^1^*% (counts/n)*	19.4% (6/31)	8.7% (2/23)
***AT % (counts/n)***	22.5% (9/40)	20.9% (9/43)
*Stroke % (counts/n)*	66.7% (6/9)	66.7% (6/9)
*MI % (counts/n)*	33.3% (3/9)	11.1% (1/9) *^2^
*PAD % (counts/n)*	0.0% (0/9)	22.2% (2/9)
***ATE and VTE % (counts/*n*)***	0.0% (0/40)	25.6% (11/43) *^3^

BMI: body mass index; VT: venous thromboembolism; AT: arterial thromboembolism; DVT: deep vein thrombosis; PE: pulmonary embolism; MI: myocardial infarction; PAD: peripheral arterial disease. *^1^ Other: arm vein thrombosis, mesenteric vein thrombosis, portal vein thrombosis, renal vein thrombosis, sinus vein thrombosis, ocular vein thrombosis, penile vein thrombosis. *^2^ the patient also suffered from peripheral arterial disease. *^3^ including stroke, PAD, MI, renal artery occlusion, PE, DVT, and arm vein thrombosis.

**Table 2:** Detection of PF4-Abs in the different anti-PF4 assays in Group 1 (patients with acute thromboembolism) and Group 2 (patients with chronic thromboembolic APS).

		PF4-Abs (IgG) Anti-PF4 IgG Prototype Chemiluminescence Assay	PF4/H-Abs (IgG) Anti-PF4/Polyanion IgG Chemiluminescence Assay *^1^	PF4/H-Abs (IgG) Anti-PF4/Heparin IgG ELISA Assay *^2^	PF4/H-Abs (IgG/IgM/IgA) Anti-PF4/Polyanion IgG/IgM/IgA Latex Agglutination Assay *^3^
		positive results in % (counts/*n*)	positive results in % (counts/*n*)	positive results in % (counts/*n*)	positive results in % (counts/*n*)
***Cut-off***		<2.03 U/mL	<1.0 U/mL	OD <0.5	<1.0 U/mL
***Group 1: Acute thromboembolism (ATE)***	n=				
*All*	**40**	**7.5% (3/40)**	**10.0% (4/40)**	**20.0% (8/40)**	**2.5% (1/40)**
*spontaneous thromboses*	10	0.0% (0/10)	0.0% (0/10)	30.0% (3/10)	0.0% (0/10)
*infection-associated thromboses*	8	25.0% (2/8)	25.0% (2/8)	12.5% (1/8)	0.0% (0/8)
*vaccine-associated thromboses*	2	0.0% (0/2)	0.0% (0/2)	0.0% (0/2)	0.0% (0/2)
*recurrent thromboses despite * *anticoagulation*	20	5.0% (1/20)	10.0% (2/20)	20.0% (4/20)	5.0% (1/20)
***Group 2: APS patients with chronic thromboembolic disease (APS-CTE)***	**43**	**11.6% (5/43)**	**11.6% (5/43)**	**16.3% (7/43)**	**2.3% (1/43)**

PF4-Abs: anti-PF4 antibodies; PF4/H-Abs: anti-PF4/heparin antibodies; *^1^ HemosIL^®^ AcuStar HIT-IgG_(PF4-H)_; *^2^ ZYMUTEST^®^ HIA IgG; *^3^ HemosIL^®^ HIT-Ab_(PF4-H)_; U/ml: units per milliliter, OD: optical density.

**Table 3:** Group 1: Individual results of patients with acute thromboembolism with at least one positive PF4-Abs result (ATE; 27.5%: 11/40).

Patient Number	PF4-Abs (IgG) Anti-PF4 IgG Prototype Chemiluminescence Assay	PF4/H-Abs (IgG) Anti-PF4/ Polyanion IgG Chemiluminescence Assay *^1^	PF4/H-Abs (IgG) Anti-PF4/Heparin IgG ELISA Assay *^2^	PF4/H-Abs (IgG/IgM/IgA) Anti-PF4/Polyanion IgG/IgM/IgA Latex Agglutination Assay *^3^	Exposition with Heparin (UFH or LMWH)	Time Between Start of Heparin Exposure and Blood Draw	Duration of Heparin Exposure	Platelet Count
	Cut-off <2.03 U/mL	Cut-off <1.0 U/mL	Cut-off OD < 0.5	Cut-off <1.0 U/mL		in days	in days	Cut-off >140/nL (women) >151/nL (men)
*2*	**3.42**	**1.08**	0.43	0.50	none	/	/	208
*6*	1.19	0.0	**1.22**	0.10	none	/	/	483
*8*	0.6	0.08	**0.75**	0.00	none	/	/	245
*9*	0.5	**2.48**	**0.71**	0.00	LMWH	13	4	188
*14*	0.93	0.28	0.45	**3.30**	LMWH	30	30	252
*17*	**3.72**	**1.58**	0.08	0.40	none	/	/	355
*18*	**2.88**	**1.41**	**0.84**	0.50	LMWH	16	5	172
*30*	1.37	0.12	**1.20**	0.4	LMWH	50	7	230
*36*	0.39	0.6	**1.04**	0.1	n.a.	n.a.	n.a.	192
*37*	0.57	0.73	**0.84**	0.80	none	/	/	249
*38*	0.29	0.03	**1.10**	0.20	none	/	/	**125**

PF4-Abs: anti-PF4 antibodies; PF4/H-Abs: anti-PF4/heparin antibodies; n.a.: not available; UFH: unfractioned heparin; LMWH: low-molecular-weight heparin. *^1^ HemosIL^®^ AcuStar HIT-IgG_(PF4-H)_; *^2^ ZYMUTEST^®^ HIA IgG; *^3^ HemosIL^®^ HIT-Ab_(PF4-H)_; U/mL: units per milliliter, OD: optical density, nL: nanoliter.

**Table 4:** Group 2: Individual results of APS patients with chronic thromboembolic disease with at least one positive PF4-Abs result (APS-CTE, 27.9%: 12/43).

Patient Number	PF4-Abs (IgG) Anti-PF4 IgG Prototype Chemiluminescence Assay	PF4/H-Abs (IgG) Anti-PF4/Polyanion IgG Chemiluminescence Assay *^1^	PF4/H-Abs (IgG) Anti-PF4/Heparin IgG ELISA Assay *^2^	PF4/H-Abs (IgG/IgM/IgA) Anti-PF4/Polyanion IgG/IgM/IgA Latex Agglutination Assay *^3^	Platelet Count
	Cut-off <2.03 U/mL	Cut-off <1.0 U/mL	Cut-off OD < 0.5	Cut-off <1.0 U/mL	Cut-off >140/nL (women)/>151/nL (men)
*1*	0.23	0.07	**0.7**	0.0	255
*3*	0.16	0.16	**0.67**	0.3	60 *
*5*	0.68	0.18	**1.23**	0.1	221
*6*	**2.34**	**1.18**	0.44	0.2	228
*9*	**6.97**	**2.66**	0.29	0.3	472
*11*	1.23	**1.1**	0.28	0.6	468
*12*	0.05	0.0	**1.06**	0.1	207
*27*	**2.09**	**3.39**	**0.76**	0.3	**129**
*28*	0.37	0.03	**1.23**	0.4	291
*35*	**3.37**	0.12	0.15	0.3	**48**
*37*	**2.57**	**1.13**	0.1	**3.5**	298
*39*	1.14	0.65	**0.63**	0.5	236

PF4-Abs: anti-PF4 antibodies, PF4/H-Abs: anti-PF4/heparin antibodies; * positive HIPA and PIPA results. *^1^ HemosIL^®^ AcuStar HIT-IgG_(PF4-H)_; *^2^ ZYMUTEST^®^ HIA IgG; *^3^ HemosIL^®^ HIT-Ab_(PF4-H)_; U/mL: units per milliliter, OD: optical density, nL: nanoliter.

## Data Availability

The data presented in this study are not publicly available because they were generated using a proprietary prototype provided by an industrial partner that is not yet commercially available. The data are subject to confidentiality restrictions and cannot be shared.

## Abbreviations

The following abbreviations are used in this manuscript:

ACR	American College of Rheumatology
APIT	Anti-PF4-associated immune thromboses
APS	Antiphospholipid syndrome
APS-CTE	APS patients with chronic thromboembolic disease
aPL	Antiphospholipid antibodies
aPTT	Activated partial thromboplastin time
ATE	Acute thromboembolism
AT	Arterial thromboembolism
BMI	Body mass index
DOAC	Direct oral anticoagulant
DVT	Deep vein thrombosis
ELISA	Enzyme-linked immunosorbent assay
EULAR	European Alliance of Associations for Rheumatology
FVL	Factor V Leiden mutation
HbA1c	Glycated hemoglobin
HDL	High-density lipoprotein
HIPA	Heparin-induced platelet activation assay
HIT	Heparin-induced thrombocytopenia
LDL	Low-density lipoprotein
LMWH	Low-molecular-weight heparin
MGTS	Monoclonal gammopathy of thrombotic significance
MI	Myocardial infarction
OD	Optical density
PAD	Peripheral arterial disease
PE	Pulmonary embolism
PF4	Platelet factor 4
PF4-Abs	Anti-PF4 antibodies
PF4/H-Abs	Anti-PF4/heparin antibodies
PI	Platelet inhibitor
PFO	Patent foramen ovale
PIPA	PF4-induced platelet activation assay
PT	Prothrombin time
SRA	Serotonin release assay
TIA	Transient ischemic attack
UFH	Unfractionated heparin
VITT	Vaccine-induced immune thrombotic thrombocytopenia
VT	Venous thromboembolism
β2-GPI	β2-glycoprotein I
